# Predictive values of neutrophil-to-lymphocyte ratio on disease severity and mortality in COVID-19 patients: a systematic review and meta-analysis

**DOI:** 10.1186/s13054-020-03374-8

**Published:** 2020-11-16

**Authors:** Xiaoming Li, Chao Liu, Zhi Mao, Minglu Xiao, Li Wang, Shuang Qi, Feihu Zhou

**Affiliations:** 1 Department of Critical Care Medicine, The First Medical Centre, Chinese People’s Liberation Army General Hospital, 28 Fu-Xing Road, Beijing, 100853 People’s Republic of China; 2 Medical School of Chinese PLA, Beijing, People’s Republic of China; 3 The Fourth Medical Centre, Chinese People’s Liberation Army General Hospital, Beijing, People’s Republic of China

**Keywords:** Neutrophil-to-lymphocyte ratio, Disease severity, Mortality, Predictive, Systematic review, Meta-analysis

## Abstract

**Background:**

Coronavirus disease 2019 (COVID-19), a highly infectious disease, has been rapidly spreading all over the world and remains a great threat to global public health. Patients diagnosed with severe or critical cases have a poor prognosis. Hence, it is crucial for us to identify potentially severe or critical cases early and give timely treatments for targeted patients. In the clinical practice of treating patients with COVID-19, we have observed that the neutrophil-to-lymphocyte ratio (NLR) of severe patients is higher than that in mild patients. We performed this systematic review and meta-analysis to evaluate the predictive values of NLR on disease severity and mortality in patients with COVID-19.

**Methods:**

We searched PubMed, EMBASE, China National Knowledge Infrastructure (CNKI) and Wanfang databases to identify eligible studies (up to August 11, 2020). Two authors independently screened studies and extracted data. The methodological quality of the included studies was assessed by Quality Assessment of Diagnostic Accuracy Studies 2 (QUADAS-2).

**Results:**

Thirteen studies involving 1579 patients reported the predictive value of NLR on disease severity. The pooled sensitivity (SEN), specificity (SPE) and area under curve (AUC) were 0.78 (95% CI 0.70–0.84), 0.78 (95% CI 0.73–0.83) and 0.85 (95% CI 0.81–0.88), respectively. Ten studies involving 2967 patients reported the predictive value of NLR on mortality. The pooled SEN, SPE and AUC were 0.83 (95% CI 0.75–0.89), 0.83 (95% CI 0.74–0.89) and 0.90 (95% CI 0.87–0.92), respectively.

**Conclusions:**

NLR has good predictive values on disease severity and mortality in patients with COVID-19 infection. Evaluating NLR can help clinicians identify potentially severe cases early, conduct early triage and initiate effective management in time, which may reduce the overall mortality of COVID-19.

**Trial registry:**

This meta-analysis was prospectively registered on PROSPERO database (Registration number: CRD42020203612).

## Introduction

Coronavirus disease 2019 (COVID-19), a highly infectious disease caused by severe acute respiratory syndrome coronavirus 2 (SARS-CoV-2), has been rapidly spreading all over the world and remains a great threat to global public health [1]. The clinical symptoms of patients with COVID-19 vary widely. A significant proportion of patients with COVID-19 have mild symptoms, such as fever, muscle ache, cough, shortness of breath and fatigue, and about half of patients do not show any obvious symptoms [2, 3]. However, some severe cases with severe pneumonia can develop into acute respiratory distress syndrome (ARDS), pulmonary oedema or multiple organ dysfunction syndrome (MODS), hence leading to a high mortality [4–6]. Although many patients have mild symptoms, they may suddenly progress to ARDS, septic shock or even MODS [7]. Patients diagnosed with severe or critical illness have a poor prognosis. Hence, it is crucial for us to identify potentially severe or critical cases early and give timely treatments for targeted patients. Therefore, we can prevent the progression of COVID-19, save medical resources and reduce mortality.

Similar to patients with Middle East respiratory syndrome (MERS) and severe acute respiratory syndrome (SARS), dysregulated inflammation leading to cytokine storms is associated with worsening clinical outcomes in patients with COVID-19 [8–10]. Emerging evidences suggested that peripheral blood neutrophil-to-lymphocyte ratio (NLR) can be used as a marker of systemic inflammation. Furthermore, NLR has shown good predictive values on progression and clinical outcomes in various disease, such as solid tumours, chronic obstructive pulmonary disease (COPD), cardiovascular disease and pancreatitis [11–14]. Recently, several studies have reported that NLR may differentiate between mild/moderate and severe/critical groups and probability of death in patients with COVID-19 infection. In addition, a series of studies have suggested NLR is a reliable predictor of COVID-19 progression and elevated NLR is associated with high mortality [15–20].

NLR is a readily available biomarker that can be calculated from components of the differential white cell count (dividing neutrophil by lymphocyte count). We performed this systematic review and meta-analysis to evaluate the predictive values of NLR on disease severity and mortality in patients with COVID-19 and to provide a reliable marker for early identification of potentially severe or critically ill cases.

## Methods

We followed the Preferred Reporting Items for Systematic Reviews and Meta-Analyses (PRISMA statement) guidelines to perform this meta-analysis [21]. It was prospectively registered on PROSPERO database (Registration number: CRD42020203612).

### Selection of studies

We reviewed PubMed, EMBASE, China National Knowledge Infrastructure (CNKI) and Wanfang databases through August 11, 2020. The search terms were as follows: (“Neutrophil to lymphocyte ratio” or “neutrophil lymphocyte ratio” or “neutrophil-to-lymphocyte ratio” or “neutrophil/lymphocyte ratio” or “NLR”) and (“Coronavirus disease 2019” or “2019 Novel Coronavirus” or “SARS-CoV-2” or “2019-nCoV” or “COVID-19”). The detail of search strategy of PubMed is shown in Additional file 1. No language restrictions were imposed. To find additional citations, the reference lists of the included studies and recent review articles were screened when necessary.

Two authors (X.L and C.L) independently screened all identified citations to find studies for the final analysis. Any disagreement was resolved through discussion. In case of persistent disagreement, we consulted the third reviewer (F.Z) for arbitration. Studies were selected if they met the following criteria: (1) The predictive value of NLR on disease severity or mortality in patients with COVID-19 was evaluated; (2) a 2 × 2 table of results could be constructed [sufficient information to calculate true positive (TP), false positive (FP), false negative (FN) and true negative (TN)]. The exclusion criteria were as follows: (1) case report, review, editorial, conference abstract, comment, letter, animal study; (2) unable to extract a 2 × 2 table of results.

### Data extraction and quality assessment

Two authors (X.L and C.L) independently extracted relevant information from individual studies, including first author, publication year, country, publication language, number of patients (male/female), mean age, cut-off value, area under curve (AUC), TP, TN, FP, FN, sensitivity (SEN) and specificity (SPE). The extracted information was checked by a third author (Z.M). We used the Quality Assessment of Diagnostic Accuracy Studies 2 (QUADAS-2) criteria to evaluate each of the included studies in 4 domains: patient selection; index test; reference standard; and flow and test timing [22].

### Statistical analysis

The statistical analyses were conducted by STATA (version 14.0) using MIDAS module [23]. A bivariate random-effects regression model was performed to calculate SEN, SPE, positive likelihood ratio, negative likelihood ratio, diagnostic odds ratio (DOR) and corresponding 95% credible interval (CI). A summary receiver operating characteristic (SROC) curve was drawn to assess the overall diagnostic accuracy. The higher the AUC value, the better the diagnostic power [24]. We used Deek funnel plot to detect publication bias. If the P value is less than 0.1, publication bias may exist. I^2^ index was calculated to assess heterogeneity between studies, and I^2^ values above 50% were regarded as the indicative of substantial heterogeneity [25]. We conducted Fagan nomograph to explore the relationship between the pre-test probability, likelihood ratio and the post-test probability. To investigate potential sources of heterogeneity among included studies, sensitivity and subgroup analyses were conducted. In sensitivity analyses, we only included studies published in English. We did subgroup analyses based on cut-off value.

## Results

### Selection and characteristics of studies

**Fig. 1:**
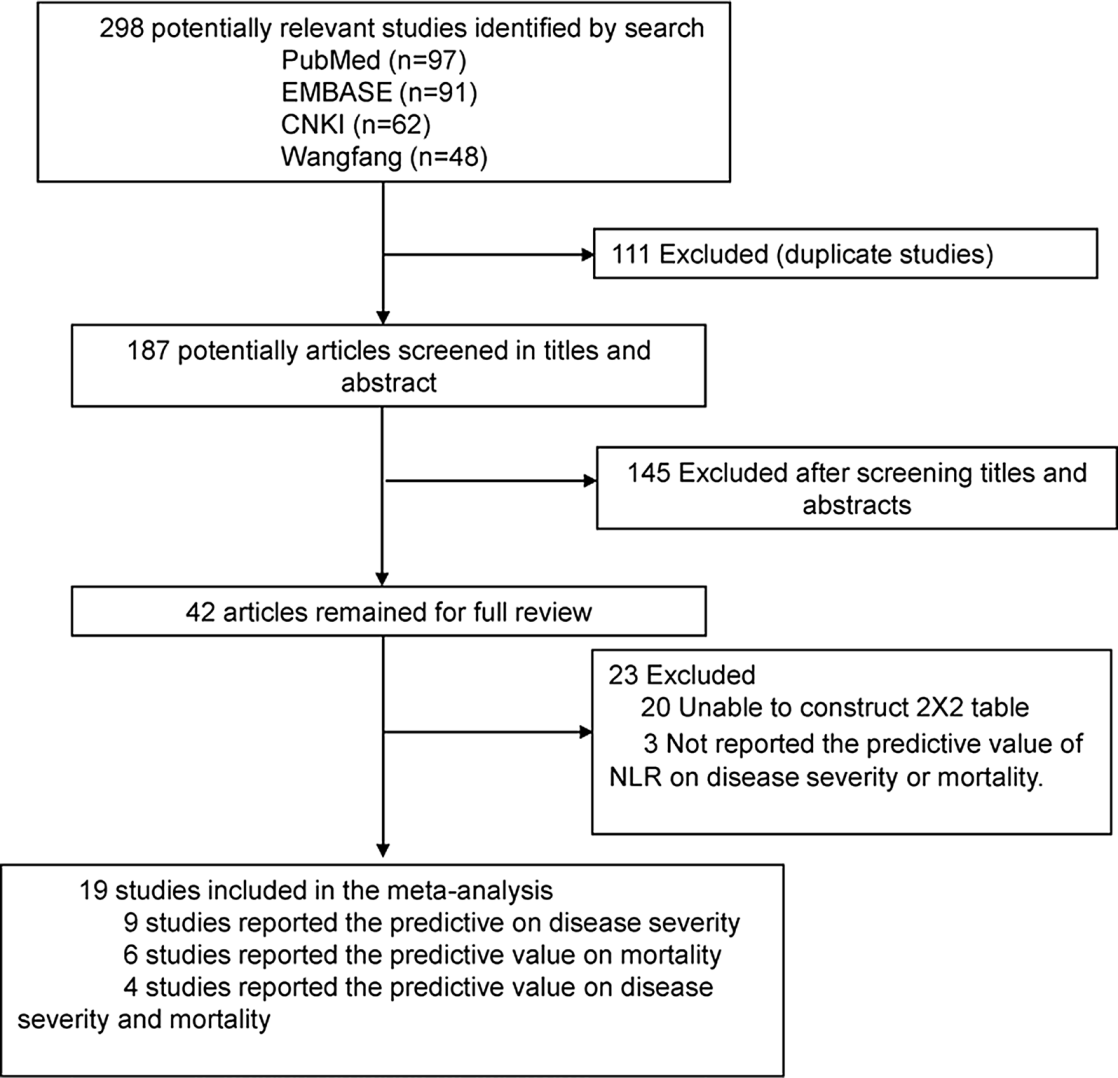
Flow diagram for the identification of eligible studies

As a result of the literature search, a total of 298 studies were identified, including 97 from PubMed, 91 from EMBASE, 62 from CNKI and 48 from Wanfang debase. Figure 1 shows the study selection process. In total, 111 duplicate publications were excluded. According to the inclusion and exclusion criteria, we excluded 145 studies by evaluating the titles and abstracts. The remaining 42 studies were further scrutinized by reading the full text. Finally, only 19 studies were included in this meta-analysis, of which 9 reported the predictive value on disease severity [26–34], 6 reported the predictive value on mortality [35–40], and 4 reported the predictive value on both disease severity and mortality [41–44].

The characteristics of the included studies and the predictive value of NLR on disease severity or mortality in each study are presented in Table 1. Most studies were conducted in China. Twelve studies were published in English, six in Chinese and one in Spanish. Except one prospective study [27], all others were retrospective studies. The number of participants across studies ranged from 45 to 1004. Notably, the SEN, SPE, AUC and cut-off value of NLR predicting mortality and disease severity ranged greatly among the included studies. Except two studies [41, 42], all other studies defined severe patients as meeting at least one of the following criterions: shortness of breath, respiratory rate (RR) ≥ 30 times/min or oxygen saturation (resting state) ≤ 93%, or PaO2/FiO2 ≤ 300 mmHg.

**Table 1:** Characteristics of the included studies and diagnostic test performance of NLR for disease severity and mortality

	Study	Country	Publication language	No. of patients	Male/ female	Mean age	Cut-off	AUC	TP	FP	FN	TN	SEN (%)	SPE (%)
Severity	Yang2020 [33]	China	English	93	56/37	46.4 ± 17.6	3.3	0.84	21	25	3	44	88.0	63.6
	Wang2020 [31]	China	English	45	23/22	39.0 ± 11.5	13.4	0.89	8	6	2	29	83.3	82.4
	Fesih2020 [44]	Turkey	English	139	62/77	55.5 ± 18.5	3.3	0.87	43	25	11	60	79.0	71.0
	Jingyuan Liu2020 [27]	China	English	115	64/51	NA	3.1	NA	28	13	9	65	75.7	83.3
	Asghar2020	Pakistan	English	100	69/31	52.6 ± 15.7	3.7	0.80	29	25	4	42	87.9	62.1
	Sun2020 [30]	China	English	116	60/56	50.0 ± 4.0	4.5	0.89	20	9	7	80	74.1	89.9
	Shang2020 [29]	China	English	443	220/223	56.0 ± 17.4	4.3	0.74	78	50	61	254	56.3	83.7
	Yueping Liu2020 [28]	China	English	84	47/37	53.0 ± 17.8	4.9	0.76	13	8	10	53	56.5	86.9
	Basbus2020 [42]	Spain	Spanish	131	71/60	52.0 ± 30.4	3.0	NA	17	36	4	74	80.9	67.3
	Li2020 [43]	China	Chinese	93	55/38	62.1 ± 16.8	11.3	NA	34	4	9	46	79.1	92.0
	Zha2020 [34]	China	Chinese	85	57/28	54.2 ± 16.0	5.6	0.77	25	10	12	38	68.8	78.4
	Fei2020 [26]	China	Chinese	72	32/40	58.0 ± 13.8	3.0	0.89	20	14	0	38	100	73.1
	Xia2020 [32]	China	Chinese	63	33/30	63.4 ± 14.9	4.8	0.83	26	8	5	24	83.9	75.0
Mortality														
	Cheng2020 [36]	China	English	456	211/245	55.0 ± 18.6	3.2	0.81	26	107	10	303	78.3	73.9
	Tatum2020 [38]	America	English	125	57/68	58.7 ± 14.8	10.0	0.71	12	4	11	98	52.4	96.7
	Chen2020 [35]	China	English	681	362/219	65.0 ± 13.3	6.7	0.86	87	130	17	447	83.7	77.5
	Fesih2020 [44]	Turkey	English	139	62/77	55.5 ± 18.5	5.7	0.85	11	13	2	113	83.0	90.0
	Asghar2020 [41]	Pakistan	English	100	69/31	52.6 ± 15.7	4.2	0.81	20	29	2	49	90.9	62.6
	Yan2020 [39]	China	English	1004	493/511	NA	11.8	0.95	39	211	1	753	97.5	78.1
	Basbus2020 [42]	Spain	Spanish	131	71/60	52.0 ± 30.4	3	NA	7	46	2	76	77.8	62.3
	Li2020 [43]	China	Chinese	93	55/38	62.1 ± 16.8	11.3	0.92	28	10	3	52	90.3	83.9
	Song2020 [37]	China	Chinese	84	56/28	66.5 ± 12.2	6.1	0.87	32	5	10	37	76.2	88.1
	Zhang2020 [40]	China	Chinese	154	81/73	69.2 ± 7.5	9.4	0.86	21	10	6	117	76.2	92.0

*AUC* area under curve, *TP* true positive, *FP* false positive, *FN* false negative, *TN* true negative, *SEN* sensitivity, *SPE* specificity, *NLR* neutrophil-to-lymphocyte ratio, *NA* not available

### Study quality and publication bias

The methodological quality of the included studies is presented in Additional file 2. One study only included patients classified as moderate [36], one included only severe patients [35], and another included only elderly patients [40]. Therefore, these three studies were considered to have a high risk of patient selection bias. One study included 32 moderate cases, and another 31 severe cases were included as a control group [32]. One study included 48 moderate cases, and another 37 severe cases were included as a control group [34]. One study included 50 moderate cases, and another 43 severe cases were included as a control group [43]. One study included 42 dead patients, and another 42 discharged patients were included as a control group [37]. These four studies were also assessed to show high risk of patient selection bias, because they did not avoid a case–control design. One study did not provide sufficient information about patients enrolled and leaded to a high risk of patient selection in our opinion [33]. Most studies were considered to have unclear risk of bias regarding index tests, because they did not report the blindness between index and reference tests. Deek funnel plot is shown in Additional file 3, and publication bias may exist among studies reporting the predictive value of NLR on disease severity (P = 0.04).

### Predictive value of NLR on disease severity

**Fig. 2:**
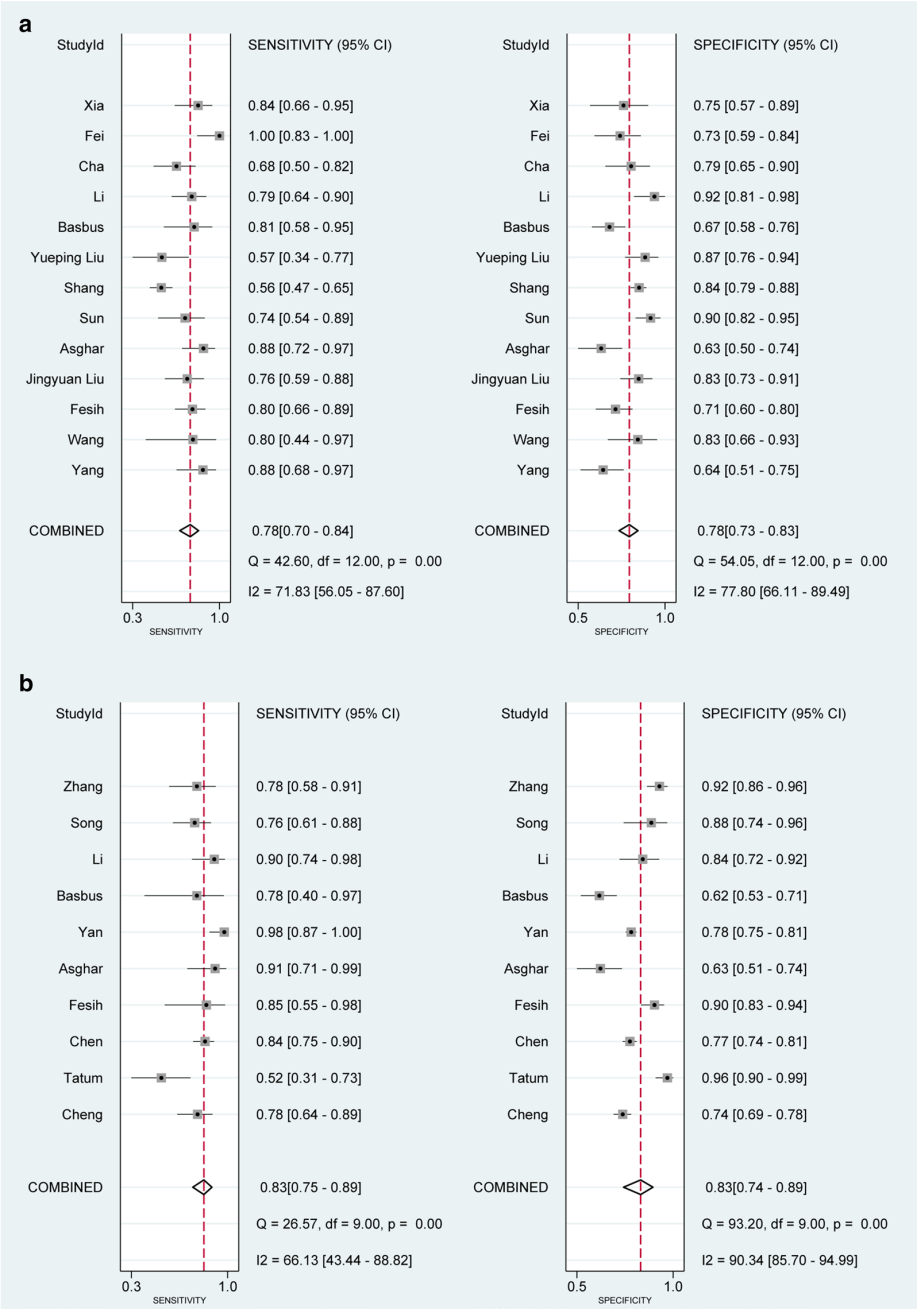
**a.** Forest plot of the sensitivity and specificity of NLR for predicting disease severity in patients with COVID-19. The pooled sensitivity and specificity were 0.78 (95% CI 0.70–0.84) and 0.78 (95% CI 0.73–0.83), respectively. **b.** Forest plot of the sensitivity and specificity of NLR for predicting mortality in patients with COVID-19. The pooled sensitivity and specificity were 0.83 (95% CI 0.75–0.89) and 0.83 (95% CI 0.74–0.89), respectively

**Fig. 3:**
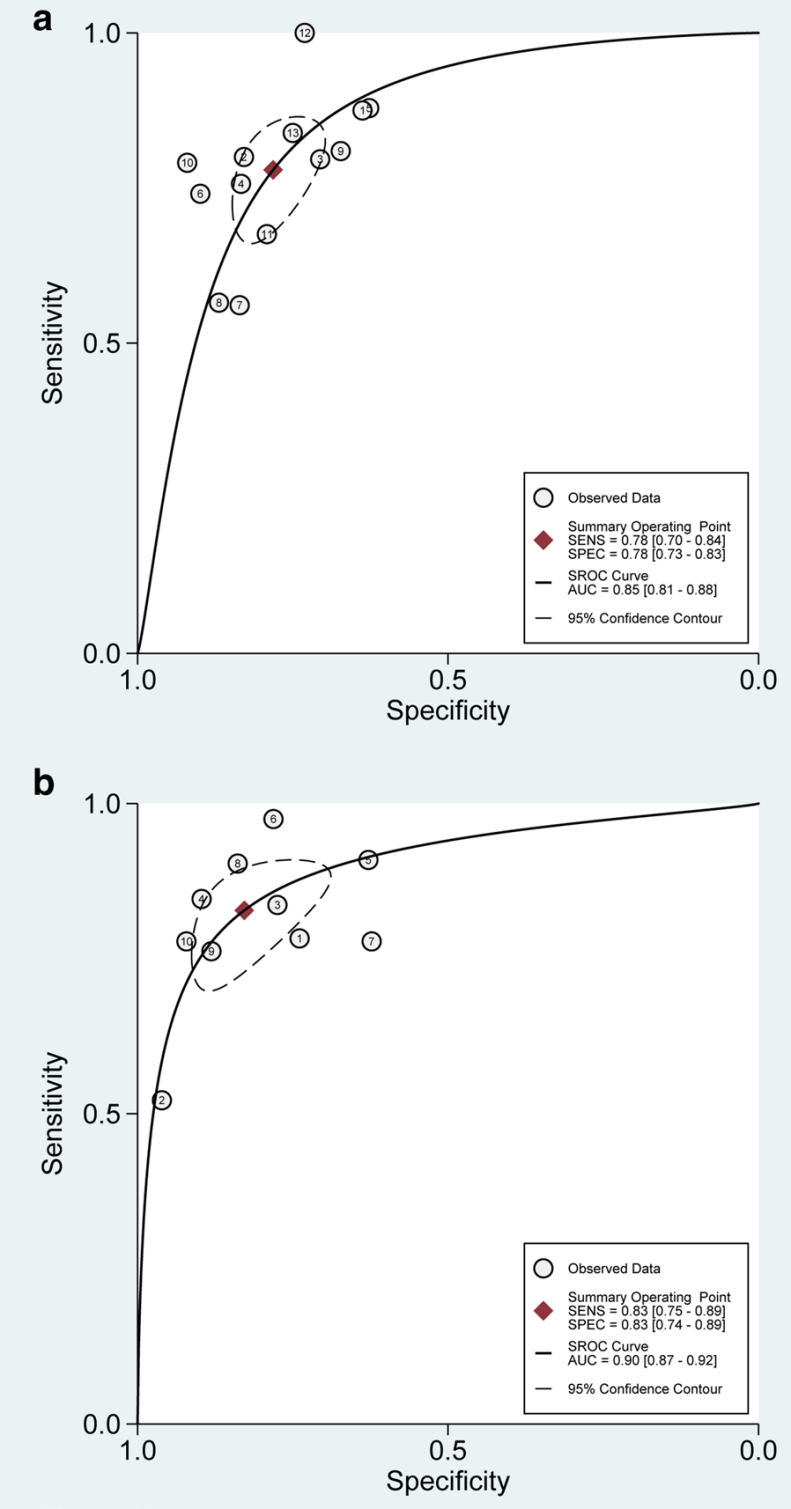
Summary receiver operating characteristic graph for the included studies. **a.** The AUC of NLR for predicting disease severity was 0.85 (95% CI 0.81–0.88). **b.** The AUC of NLR for predicting mortality was 0.90 (95% CI 0.87–0.92)

**Fig. 4:**
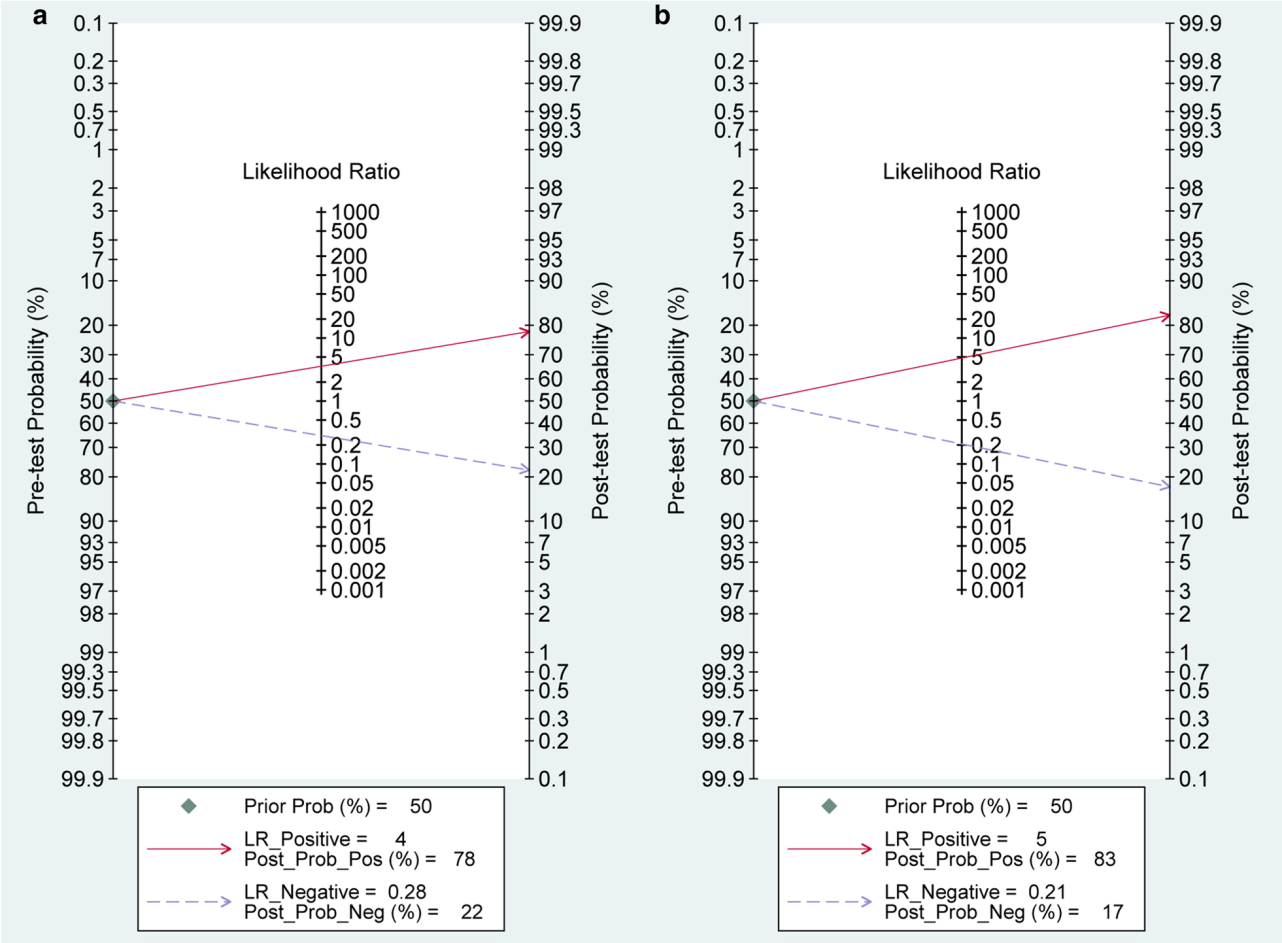
Fagan nomogram of NLR for predicting disease severity and mortality in patients with COVID-19. The pre-test probability was set to 50%. **a.** The post-test probability of NLR for the detection of severe cases was 78% when the NLR was above the cut-off value. The post-test probability was 22% when the NLR was below the cut-off value. **b.** The post-test probability of NLR for the detection of mortality was 83% when the NLR was above the cut-off value. The post-test probability was 17% when the NLR was below the cut-off value

Thirteen studies involving 1579 patients reported the predictive value of NLR on disease severity. The pooled SEN and SPE were 0.78 (95% CI 0.70–0.84, I^2^ = 71.83) and 0.78 (95% CI 0.73–0.83, I^2^ = 77.80), respectively (Fig. 2a). The positive likelihood ratio was 3.6 (95% CI 2.9–4.4), and the negative likelihood ratio was 0.28 (95% CI 0.21–0.38). The DOR was 13 (95% CI 9–18). The SROC curve is shown in Fig. 3a. The AUC of NLR for predicting disease severity was 0.85 (95% CI 0.81–0.88), indicating high diagnostic value. We can learn from Fagan nomogram (Fig. 4a) that if the pre-test probability was set to 50%, the post-test probability of NLR for the detection of severe cases was 78% when the NLR was above the cut-off value. On the contrary, when the NLR was below the cut-off value, the post-test probability was 26%.

### Predictive value of NLR on mortality

Ten studies involving 2967 patients reported the predictive value of NLR on mortality. The pooled SEN and SPE were 0.83 (95% CI 0.75–0.89, I^2^ = 66.13) and 0.83 (95% CI 0.74–0.89, I^2^ = 90.34), respectively (Fig. 2b). The positive likelihood ratio was 4.8 (95% CI 3.3–7.0), and the negative likelihood ratio was 0.21 (95% CI 0.15–0.30). The DOR was 23 (95% CI 15–36). The SROC with pooled diagnostic accuracy was 0.90 (95% CI 0.87–0.92), presented in Fig. 3b. The Fagan nomogram showed that the post-test probability of NLR for the detection of mortality was 83% when the NLR was above the cut-off value and the post-test probability was 17% when the NLR was below the cut-off value (Fig. 4b).

### Subgroup analyses and sensitivity analyses

We conducted the subgroup analyses based on the cut-off value. In terms of predicting disease severity, the cut-off value in six studies was higher than 4.5 and was termed the “high cut-off value” subgroup. Seven others used a lower cut-off value, which were included in the “low cut-off value” subgroup. The AUC was 0.86 (95% CI 0.83–0.89) and 0.82 (95% CI 0.78–0.85), respectively. Similarly, ten studies reporting the predictive value of NLR on mortality were divided into “high cut-off value” (cut-off ≥ 6.5) and “low cut-off value” (< 6.5) subgroups, and the AUC was 0.92 (95% CI 0.89–0.94) and 0.84 (95% CI 0.80–0.87), respectively. In the sensitivity analyses, we only included studies published in English. The pooled AUC for predicting disease severity and mortality was 0.83 (95% CI 0.80–0.86) and 0.90 (95% CI 0.87–0.92), respectively. Detailed results about subgroup analyses and sensitivity analyses are presented in Table 2.

**Table 2:** Results of sensitivity analysis and subgroup analysis

Categories	No. of studies	Sensitivity (95% CI)/I^2^	Specificity (95% CI) /I^2^	AUC (95% CI)	DOR (95% CI)	PLR/NLR
*Disease severity*						
Cut-off ≥ 4.5	6	0.74(0.66,0.80)/25.56	0.86(0.81,0.89)/36.40	0.86(0.83,0.89)	17(10,28)	5.1/0.31
Cut-off < 4.5	7	0.82(0.71,0.89)/82.74	0.72(0.66,0.78)/79.70	0.82(0.78,0.85)	12(7,19)	3.0/0.25
Published in English	8	0.74(0.63,0.83)/73.82	0.78(0.71,0.84)/81.99	0.83(0.80,0.86)	10(7,16)	3.4/0.33
*Mortality*						
Cut-off ≥ 6.5	5	0.83(0.66,0.92)/84.97	0.87(0.77,0.93)/92.60	0.92(0.89,0.94)	32(17,61)	6.3/0.20
Cut-off < 6.5	5	0.81(0.72,0.87)/0	0.77(0.64,0.86)/89.07	0.84(0.80,0.87)	14(7,27)	3.5/0.25
Published in English	6	0.83(0.69,0.91)/79.98	0.82(0.71,0.90)/89.87	0.90(0.87,0.92)	23(12,41)	4.7/0.21

*AUC* area under curve, *PLR* positive likelihood ratio, *NLR* negative likelihood ratio, *DOR* diagnostic odds ratio, *CI* credible interval

## Discussion

Although in the clinical practice of treating patients with COVID-19, we have observed that the NLR of severe patients is higher than that in mild patients, there is no systematic review and meta-analysis to evaluate the predictive values of NLR on disease severity and mortality in patients with COVID-19. Studies have reported various thresholds to NLR. Clinicians are therefore unclear regarding the thresholds of NLR that should be applied in order to categorize severity of disease and predict prognosis. Our study suggested that NLR can not only be a good biomarker predicting disease severity in patients with COVID-19 (AUC = 0.85, SEN = 0.78 and SPE = 0.78), but also have value in predicting mortality (AUC = 0.90, SEN = 0.83 and SPE = 0.83).

COVID-19 spread rapidly and is an ongoing global pandemic. Medical workers from different countries make efforts to explore the best diagnostic method and the most effective treatment for COVID-19. More and more studies have focused on COVID-19 and published in different languages. To find enough studies that reported the predictive values of NLR on disease severity and mortality in patients with COVID-19, we did not impose any language restrictions. In our final analyses, twelve studies were published in English, six in Chinese and one in Spanish. To our knowledge, English is the most widely used language in the world. Studies published in English may have a wider readership and receive peer review from different countries, while studies published in other languages may be available only to native speakers. Therefore, we performed sensitivity analyses by omitting studies not published in English. The results of the sensitivity analyses were in accordance with the main analyses, indicating that the publish language was not a confounding factor.

To our knowledge, the treatments for mild cases and severe cases are greatly different. For mild cases, there is no need to intervene too much. Some patients can even recover without any treatments. However, for severe cases, even we take many kinds of measures, such as mechanical ventilation, extracorporeal membrane oxygenation (ECMO) and continuous renal replacement therapy (CRRT), the mortality is still high [45, 46]. Therefore, if the potentially severe cases were identified early and effective treatments were taken to prevent the progression of those patients, more patients’ lives may be saved.

The current criteria for classifying mild cases and severe cases are mainly based on RR, oxygen saturation and PaO2/FiO2. These indicators are important but lack specificity for COVID-19. In laboratory examination of patients with COVID-19, the absolute value of peripheral white blood cells is usually normal or low, and lymphopenia is common [47]. However, in severe or non-survival patients with COVID-19, the lymphocytes count decreases progressively, while the neutrophils count gradually increases. This may be due to excessive inflammation and immune suppression caused by SARS-CoV-2 infection. On the one hand, neutrophils are generally regarded as pro-inflammatory cells with a range of antimicrobial activities, which can be triggered by virus-related inflammatory factors, such as interleukin-6 and interleukin-8 [9]. On the other hand, systematic inflammation triggered by SARS-CoV-2 significantly depresses cellular immunity, leading to a decrease in CD3 + T cells, CD4 + T cells and CD8 + T cells. In addition, SARS-CoV-2-infected T cells may also cause cytopathic effects on T cells [10, 48–50]. Therefore, NLR, a cost-effective marker, can be easily calculated from peripheral blood routine tests and may be associated with the progression and prognosis of COVID-19. To date, four meta-analyses have reported that patients with severe COVID-19 infection had a higher NLR than those with non-severe COVID-19 infection [51–54]. However, none of them evaluated the predictive values of NLR on disease severity and mortality.

There are several limitations in this meta-analysis. First, all but one of the studies were retrospective, meaning the data were prone to confounding factors. Second, the progression and prognosis of disease were influenced by many factors, such as age, sex and comorbidities, while we did not evaluate other factors. Finally, there was considerable heterogeneity among the included studies. Although we conducted sensitivity and subgroup analyses, the heterogeneity was not significantly decreased. That may be caused by different cut-off values, different conditions of patients or different comorbidities among the included studies. Additional high-quality studies are required to shed light on the role of NLR in the progression and prognosis of COVID-19 and find the optimal cut-off value.

## Conclusions

NLR has good predictive values on disease severity and mortality in patients with COVID-19 infection. Evaluating NLR can help clinicians identify potentially severe cases early, conduct early triage and initiate effective management in time, which may reduce the overall mortality of COVID-19.

## Supplementary information

**Additional file 1**. Search strategy terms and results of PubMed.

**Additional file 2**. Summary of the methodological quality of the studies according to the QUADAS-2 (Quality Assessment of Diagnostic Accuracy Studies-2) criteria.

**Additional file 3**. Deek funnel plot asymmetry test for publication bias, with P < 0.1 indicating publication bias.

## Data Availability

All data generated or analysed during this study are included in this published article [and its supplementary information files].

## Abbreviations

COVID-19: Coronavirus disease 2019
NLR: Neutrophil-to-lymphocyte ratio
CNKI: China National Knowledge Infrastructure
QUADAS-2: Quality Assessment of Diagnostic Accuracy Studies 2
SEN: Sensitivity
SPE: Specificity
AUC: Area under curve
SARS-CoV-2: Severe acute respiratory syndrome coronavirus 2
ARDS: Acute respiratory distress syndrome
MODS: Multiple organ dysfunction syndrome
MERS: Middle East respiratory syndrome
SARS: Severe acute respiratory syndrome
COPD: Chronic obstructive pulmonary disease
PRISMA statement: Preferred Reporting Items for Systematic Reviews and Meta-Analyses
TP: True positive
FP: False positive
FN: False negative
TN: True negative
DOR: Diagnostic odds ratio
CI: Credible interval
SROC: Summary receiver operating characteristic
RR: Respiratory rate
ECMO: Extracorporeal membrane oxygenation
CRRT: Continuous renal replacement therapy
