# Supplementary material for: Predictive values of neutrophil-to-lymphocyte ratio on disease severity and mortality in COVID-19 patients: a systematic review and meta-analysis
Source: Crit Care. 2020 Nov 16;24:647. doi: 10.1186/s13054-020-03374-8 (PMC7667659; doi:10.1186/s13054-020-03374-8)
Supplement: Supplementary file 1 — Additional file 1. Search strategy terms and results of PubMed. [file 13054_2020_3374_MOESM1_ESM.docx]

**Search strategy terms and results**

| PUBMED, searched 11 August 2020 | | |
| --- | --- | --- |
| 1 | Neutrophil to lymphocyte ratio [tiab] OR neutrophil lymphocyte ratio [tiab] OR neutrophil-to-lymphocyte ratio [tiab] neutrophil/lymphocyte ratio [tiab] NLR [tiab] | 8,662 |
| 2 | Coronavirus disease 2019 [tiab] OR 2019 Novel Coronavirus [tiab] OR SARS-CoV-2 [tiab]  OR 2019-nCoV [tiab] OR COVID-19 [tiab] | 39,238 |
| 3 | #1 AND #2 | 97 |
