# Supplementary figures and images for: Predictive values of neutrophil-to-lymphocyte ratio on disease severity and mortality in COVID-19 patients: a systematic review and meta-analysis
Source: Crit Care. 2020 Nov 16;24:647. doi: 10.1186/s13054-020-03374-8 (PMC7667659; doi:10.1186/s13054-020-03374-8)

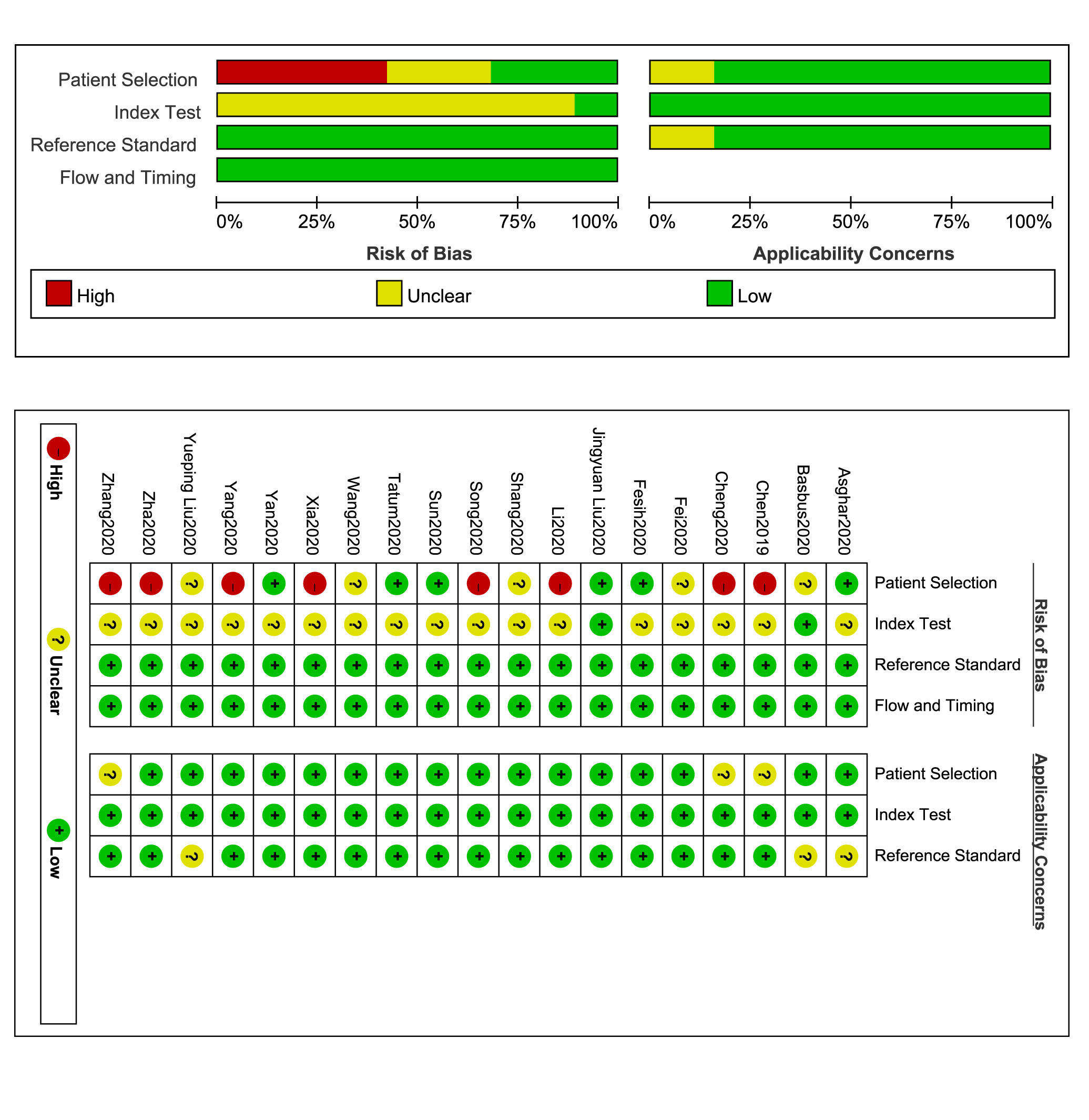

Supplement: Supplementary file 2 — Additional file 2. Summary of the methodological quality of the studies according to the QUADAS-2 (Quality Assessment of Diagnostic Accuracy Studies-2) criteria. [file 13054_2020_3374_MOESM2_ESM.tif]

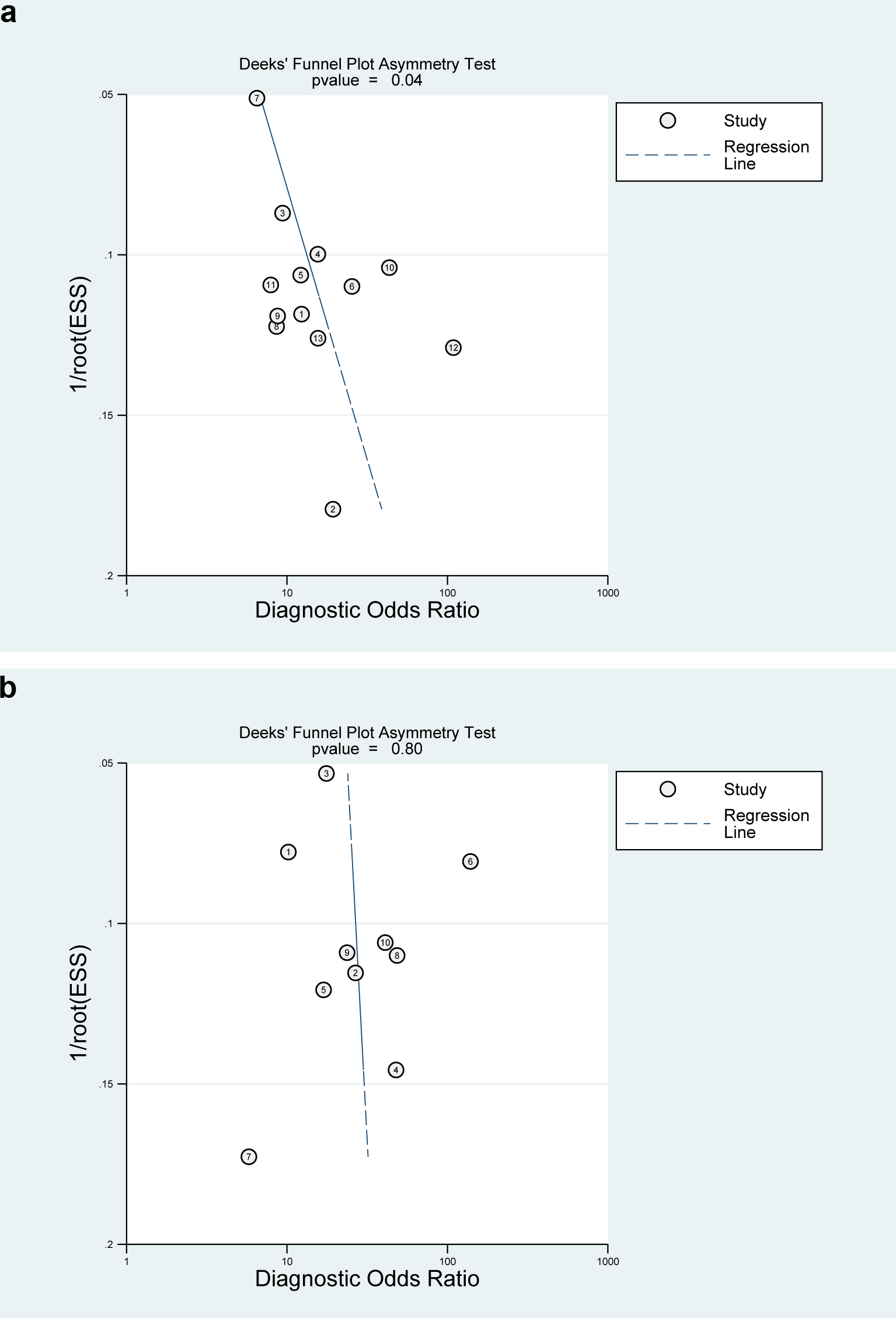

Supplement: Supplementary file 3 — Additional file 3. Deek funnel plot asymmetry test for publication bias, with P < 0.1 indicating publication bias. [file 13054_2020_3374_MOESM3_ESM.tif]
